# Predictors of Postoperative Acute Kidney Injury after Coronary Artery Bypass Graft Surgery

**DOI:** 10.21470/1678-9741-2017-0251

**Published:** 2018

**Authors:** Christian Ortega-Loubon, Manuel Fernández-Molina, Lucía Pañeda-Delgado, Pablo Jorge-Monjas, Yolanda Carrascal

**Affiliations:** 1 Department of Cardiac Surgery, Clinic University Hospital of Valladolid, Valladolid, Spain.; 2 Department of Anesthesia, Clinic University Hospital of Valladolid, Valladolid, Spain.

**Keywords:** Acute Kidney Injury, Coronary Artery Bypass, Risk Assessment, Risk Factors, Calcium Channel Blockers

## Abstract

**Objective:**

The aims of this study were to identify the risk factors associated with acute kidney injury (AKI) after isolated surgical revascularization with cardiopulmonary bypass and to develop a model to predict the appearance of postoperative AKI.

**Methods:**

A total of 435 adult patients who underwent primary isolated coronary artery bypass graft (CABG) surgery, from 2012 to 2016, in the Clinic University Hospital of Valladolid (Spain) were enrolled. AKI was defined according to the risk, injury, failure, loss, and end-stage (RIFLE) criteria. Data were collected from hospital electronic medical records. Multiple logistic regression analysis was used to identify risk factors.

**Results:**

The prevalence of AKI was 12.4%. Multivariate analysis identified age (odds ratio [OR], 1.056; 95% confidence interval [CI], 1.016-1.098; *P*=0.005), hypertension (OR, 3.078; 95% CI, 1.151-8.230; *P*=0.018), low ejection fraction (EF) (OR, 6.785; 95% CI, 2.080-22.135; *P*=0.001), estimated glomerular filtration rate (eGFR) (OR, 1.017; 95% CI, 1.005-1.028; *P*=0.014), EuroSCORE II (OR, 1.049; 95% CI, 1.004-1.096; *P*=0.033), and no intake of calcium-channel blockers (CCB) (OR, 4.892; 95% CI, 1.496-16.025; *P*=0.022) as risk factors for AKI. These risk factors were included in a model to predict postoperative AKI with an area under a receiver operating characteristic curve of 0.783±0.036 (95% CI, 0.713-0.854; *P*<0.0001).

**Conclusion:**

Age, hypertension, low EF, eGFR, EuroSCORE II, and no intake of CCB were independent risk factors for postoperative AKI. These factors provide an easy and accurate model to predict postoperative AKI in patients undergoing cardiac surgery.

**Table:** 

Abbreviations, acronyms & symbols				
ACEF	= Age, creatinine, ejection fraction		eGFR	= Estimated glomerular filtration rate
AKI	= Acute kidney injury		Hct	= Hematocrit
AKICS	= Acute kidney injury following cardiac surgery		ICU	= Intensive care unit
BMI	= Body mass index		IQR	= Interquartile range
CABG	= Coronary artery bypass graft		NYHA	= New York Heart Association
CAD	= Coronary artery disease		OR	= Odds ratio
CCB	= Calcium-channel blockers		RIFLE	= Risk, injury, failure, loss, and end-stage
CI	= Confidence interval		ROC	= Receiver operating characteristic
COPD	= Chronic obstructive pulmonary disease		RRT	= Renal replacement therapy
CPB	= Cardiopulmonary bypass		SCr	= Serum creatinine
EF	= Ejection fraction		SRI	= Simplified renal index

## INTRODUCTION

The development of postoperative acute kidney injury (AKI) is a recognized complication in patients undergoing cardiac surgery^[1]^. It has a dramatic impact on operative mortality, intensive care unit (ICU) resources, and hospital length of stay. Approximately 20% of cardiac surgical patients will develop postoperative AKI^[2]^. The risk of AKI increases 4.8 fold for each 88 µmol/L increment in serum creatinine (SCr) levels^[3-5]^. AKI severe enough to require renal replacement therapy (RRT) is infrequent, but the operative mortality in these patients ranges from 40% to 80%^[6]^. This emphasizes the crucial importance of taking any step possible to preserve renal function in the perioperative period, especially in patients at increased risk^[3,7-9]^.

The pathogenesis of AKI after cardiac surgery is not completely understood. It is very unlikely that a single etiologic factor will cause postoperative renal injury^[3]^. AKI is the consequence of multiple kidney aggressions occurring during the preoperative, intraoperative, and postoperative periods^[3]^.

Coronary artery disease (CAD) is one of the main causes of death in developed countries and coronary artery bypass graft (CABG) is the most common cardiovascular procedure performed worldwide^[10,11]^. Every interventional approach has a different impact on the onset of renal injury, but a greater risk of renal dysfunction is inevitable after cardiopulmonary bypass (CPB)^[12]^. Unlike valve surgery, which is an independent risk factor for postoperative AKI, coronary surgery is not directly related to renal dysfunction^[13]^.

Despite the fact that multiple AKI risk factors have been already identified, postoperative AKI still remains as the strongest risk factor for death after cardiac surgery^[1]^. Various predictive models have been developed to predict AKI, such as the acute kidney injury following cardiac surgery (AKICS) score, Cleveland Clinic score, Mehta score, and simplified renal index (SRI) score^[14]^. Nevertheless, their predictive value combining discrimination and calibration were barely satisfactory and not convincible^[14]^. Therefore, it is crucial to recognize these risk factors promptly and to develop a model to predict AKI's appearance, which allows appropriate measures to be taken to reduce its incidence.

This study aimed to identify the risk factors associated with AKI after isolated revascularization surgery with CPB and to make an accurate and predictive model for AKI after cardiac surgery. Such predictive model would be highly valuable for clinical practices because AKI is a well-established predictor of all-cause mortality in patients receiving CABG.

## METHODS

### Study Design

We performed an observational retrospective study of 435 consecutive patients undergoing CABG under CPB, from January 2012 to December 2016, at the Clinic University Hospital of Valladolid. All patients undergoing elective or urgent isolated primary CABG surgery under CPB were included. Those with prior renal insufficiency, off-pump surgery, prior cardiac intervention, or CABG combined with heart valve replacement or other surgical procedures were excluded.

The primary outcome variable was the development of AKI after cardiac procedure. AKI was defined according to the risk, injury, failure, loss, and end-stage (RIFLE) criteria^[15]^. The baseline SCr level was determined using the most recent level measured (mean of 1.0 ± 1.0 days) before surgery and the estimated glomerular filtration rate (eGFR) derived from the Cockcroft-Gault formula.

Secondary outcomes were need of RRT, ICU length of stay, and 30-day mortality.

### Clinical and Laboratory Variables

Preoperative variables were age at time of surgery, gender, weight, height, body mass index (BMI), personal history [*i.e.*, smoking, hypertension, peripheral vascular disease, diabetes mellitus, dyslipidemia, chronic obstructive pulmonary disease (COPD), and stroke], New York Heart Association (NYHA) classification, left ventricular function <30%, emergency surgery, need of RRT, long-term medical treatment, time elapsed between angiography and surgery, EuroSCORE I, EuroSCORE II, hemoglobin, hematocrit (Hct), SCr levels on preoperative laboratory testing, and eGFR.

Intraoperative variables were CPB and aortic cross-clamp times. Postoperative variables included AKI, need of RRT, days of stay at ICU, and 30-day mortality.

All data were obtained from electronic medical records and the cardiovascular surgery database at the medical center.

### Statistical Analysis

Data were analyzed using IBM SPSS Statistics for Windows, version 20.0, software (IBM Corp., Armonk, NY, USA).

Qualitative variables are reported as frequency and percentage and quantitative variables are reported as mean ± standard deviation or median (interquartile range - IQR) as appropriate.

Associations between AKI and other variables were identified using χ^2^ or Fisher's exact test for qualitative analysis; Student's t-test or Mann-Whitney U test was used for quantitative analysis, according to normality criteria.

All variables were included in multivariate logistic regression analyses; bootstrapping was performed to derive 95% confidence intervals (CI) for estimates. Multicollinearity was assessed using variance inflation factor. Logistic regression was repeated with variables that had been significant in previous analysis. The model was further developed and validated using the bootstrap approach, which is the method most widely advocated by experts for model development and internal validation^[16]^.

Accuracy was measured by the area under the receiver operating characteristic (ROC) curve analysis.

Odds ratio (OR) with 95% CI and *P*-value were reported. *P*-values <0.05 were considered statistically significant.

## RESULTS

Fifty-four (12.4%) patients developed postoperative AKI. Compared to patients without AKI, those who developed AKI were older, underwent operations in an urgent/emergency basis, and had higher frequency of hypertension, lower ejection fraction (EF), lower level of hemoglobin, lower Hct, higher levels of SCr, lower eGFR, longer CPB time, longer cross-clamp time, and shorter time elapsed between angiography and surgery (Table 1).

**Table 1:** Preoperative, intraoperative, and postoperative variables according to the presence of acute kidney injury (AKI).

Variables	n = 435 (%)	Non-AKI (381)	AKI (54)	*P*-value
Age (years)	66.8±9.2	66.2±9.1	70.8±8.6	0.001
Female	91 (20.9)	82 (21.5)	9 (16.7)	0.460
BMI (kg/m^2^)	27.7±3.8	27.6±3.8	28.5±4.0	0.114
Patients with BMI <18	3 (0.7)	2 (0.2)	1 (1.9)	0.235
Patients with BMI 18-24	108 (24.8)	100 (26.2)	8 (14.8)	
Patients with BMI 25-29	214 (49.2)	187 (49.1)	27 (50)	
Patients with BMI >30	110 (25.3)	92 (24.1)	18 (33.3)	
Smoking	247 (56.8)	215 (56.4)	32 (59.3)	0.695
Hypertension	327 (75.2)	279 (73.2)	48 (88.9)	0.013
Peripheral vascular disease	194 (44.6)	172 (45.4)	22 (40.7)	0.519
Diabetes mellitus	160 (36.8)	142 (37.3)	18 (33.3)	0.574
Dyslipidemia	295 (67.8)	261 (68.5)	34 (63.0)	0.415
COPD	33 (7.6)	28 (7.3)	5 (9.3)	0.585
Stroke	20 (4.6)	17 (4.5)	3 (5.6)	0.726
NYHA III-IV	86 (19.8)	74 (19.4)	12 (22.3)	0.291
Sinus rhythm	349 (80.2)	310 (81.4)	39 (72.2)	0.250
Severe left ventricular dysfunction (LVEF <30%)	22 (5.1)	13 (3.4)	9 (16.7)	0.001
Emergency surgery	59 (13.6)	45 (11.8)	14 (25.9)	0.005
Cardiogenic shock	10 (2.3)	5 (1.3)	5 (9.3)	0.047
AMI	47 (10.8)	36 (9.4)	11 (20.4)	1.000
Long-term treatment				
β-blockers	215 (49.4)	195 (51.2)	20 (37.0)	0.052
CCB	66 (15.2)	62 (16.3)	4 (7.4)	0.089
Diuretics	96 (22.1)	76 (19.9)	20 (37.0)	0.005
ACE inhibitors	192 (44.1)	164 (43.0)	28 (51.9)	0.223
Hemoglobin (g/L)	130±42.3	131±42.2	124±42.9	0.010
Hct (proportion of 1.0)	0.38±0.08	0.38±0.08	0.37±0.06	0.009
SCr (µmol/L)	106.1±221.0	101.7±238.7	114.0±53.0	0.001
eGFR (mL/min/m^2^)	87.8±34.3	90.3±34.2	70.3±29.2	0.001
Patients with eGFR >30 (mL/min/m^2^)	426 (97.9)	375 (98.4)	51 (94.4)	0.088
Patients with eGFR <30 (mL/min/m^2^)	9 (2.1)	6 (1.6)	3 (5.6)	
Time elapsed between angiography and surgery (days)	21.9±51.3	22.7±53.2	16.0±34.0	0.086
EuroSCORE I	6.3±7.3	5.6±6.0	11.1±12.1	0.001
EuroSCORE II	3.8±5.7	3.3±4.7	7.1±9.4	0.001
Intraoperative				
CPB time (min)	99.4±41.7	98.0±41.9	109.2±39.5	0.030
Cross-clamp time (min)	64.0±26.6	63.32±26.3	69.1±28.6	0.204
Postoperative				
RRT	4 (0.9)	0	4 (7.4)	0.001
Days of stay at ICU	2 (IQR 0-55)	2.7±2.2	7.4±9.3	0.001
30-day mortality	22 (5.1)	12 (3.1)	10 (18.5)	0.001

ACE=angiotensin-converting enzyme; AMI=acute myocardial infarction; BMI=body mass index; CCB=calcium-channel blockers; COPD=chronic obstructive pulmonary disease; CPB=cardiopulmonary bypass; eGFR=estimated glomerular filtration rate; Hct=hematocrit; ICU=intensive care unit; IQR=interquartile range; NYHA=New York Heart Association; RRT=renal replacement therapy; SCr=serum creatinine

Mean EuroSCORE I and EuroSCORE II were higher in the AKI group (11.1±12.1 and 7.1±9.4, respectively). Twenty-two (5.1%) patients died within 30 days of operation, with a significantly higher mortality rate in the AKI group than in the non-AKI group (18.5% *vs.* 3.1%; *P*=0.001). In addition, patients in the AKI group also had longer length of stay in the ICU (Table 1).

After incorporating the variables into the multiple logistic regression analysis with stepwise selection, age, hypertension, EF <30%, eGFR, and EuroSCORE II were independently associated with risk of postoperative AKI. Not taking calcium-channel blockers (CCB) medication was also a risk factor for the development of postoperative AKI (OR, 4.892; 95%CI, 1.496-16.025; *P*=0.022). These variables were then analyzed using bootstrap approach for the internal model validation for binary clinical risk prediction models (Table 2).

**Table 2:** Multiple logistic regression analysis for acute kidney injury (AKI) (validated with bootstrap approach).

Risk factors	B-coefficient	CI (95%)	SE	OR	CI (95%)	*P*-value
Age (years)	0.055	0.180-0.100	0.020	1.056	1.016-1.098	0.005
Hypertension	1.124	0.292-2.496	1.135	3.078	1.151-8.230	0.018
EF <30%	1.915	0.696-3.107	0.603	6.785	2.080-22.135	0.001
eGFR (mL/min/m^2^)	0.017	0.004-0.030	0.007	1.017	1.005-1.028	0.014
EuroSCORE II	0.048	0.011-0.127	0.030	1.049	1.004-1.096	0.033
No CCB intake	1.588	0.306-4.160	2.814	4.892	1.496-16.025	0.022
Constant	-9.031	-17.495 - -5.333	3.751	0.000	NA	0.001

CCB=calcium-channel blockers; CI=confidence interval; EF=ejection fraction; eGFR=estimated glomerular filtration rate; NA=not applicable; OR=odds ratio; SE=standard error

**Fig. 1:**
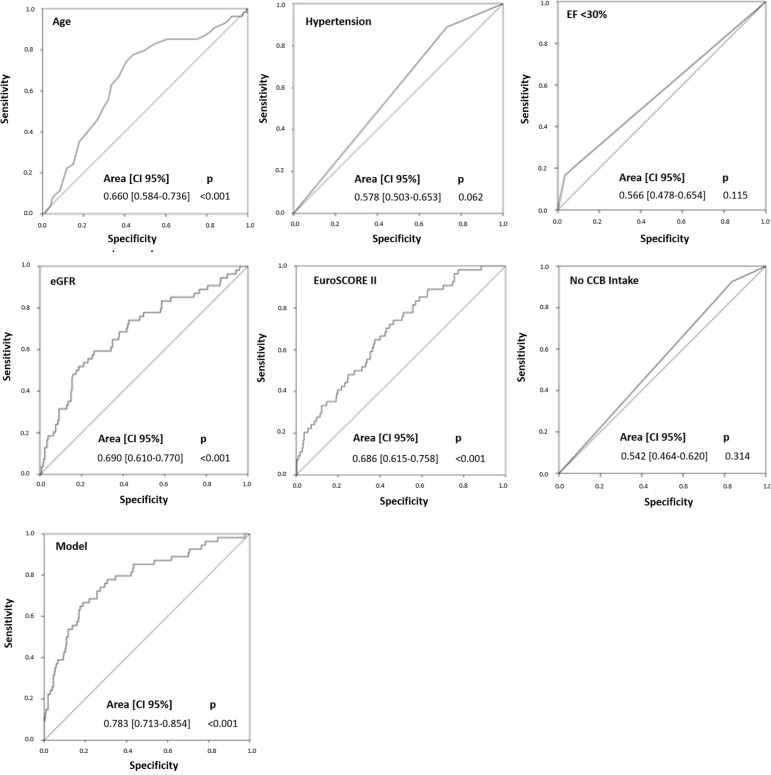
ROC curves using hypertension, EuroSCORE II, EF, eGFR, age, and CCB intake for predicting postoperative AKI. AKI=acute kidney injury; CCB=calcium-channel blockers; CI=confidence interval; EF=ejection fraction; eGFR=estimated glomerular filtration rate; ROC=receiver operating characteristic

These variables, when combined, gave us a model that accurately predicts postoperative AKI with ROC curve of 0.783±0.036 (95%CI, 0.713-0.854; *P*<0.001) (Table 3, Figure 1).

**Table 3:** Prediction model of acute kidney injury (AKI) using the risk factors resulted from the multiple logistic regression analysis.

Risk factors	ROC curve*	SE	CI (95%)	*P*-value
Age	0.660	0.039	0.584-0.736	<0.001
Hypertension	0.579	0.038	0.504-0.654	0.059
EF <30%	0.566	0.045	0.478-0.654	0.116
eGFR	0.690	0.041	0.610-0.770	<0.001
EuroSCORE II	0.686	0.036	0.615-0.758	<0.001
No CCB intake	0.542	0.040	0.464-0.620	0.314
Model	0.783	0.036	0.713-0.854	<0.001

* Hosmer-Lemeshow (H-L) value=0.720

CCB=calcium-channel blockers; CI=confidence interval; EF=ejection fraction; eGFR=estimated glomerular filtration rate; ROC=receiver operating characteristic; SE=standard error

## DISCUSSION

AKI is a well-known major complication associated with cardiac surgery and its incidence ranges from 8.9% to 42.5%, depending on the definition^[17-19]^. The incidence of AKI following CABG in our population is fairly consistent with the incidence reported in the published data, which was described from 12% to 48.5%^[19-21]^, with an associated mortality of 12.6%. Furthermore, several studies have demonstrated that 3.8%-7% of patients would require RRT^[22]^. Our reported mortality was slightly higher (18.5%) and may be related to the older age of our patient population, compared with reported series of younger patients with postoperative AKI^[20]^.

The development of AKI is tied to poor postoperative outcomes and high mortality rates in patients undergoing isolated CABG. There are multiple mechanisms involved with AKI development, including ischemic reperfusion lesion, nephrotoxin release, hemolysis, oxidative stress, and cytokine secretion, which produce systemic inflammatory responses, endothelial lesion, and tubular cell damage^[23]^.

Patients who developed AKI were older and had higher BMI, hypertension, severe left ventricular dysfunction, and worse preoperative renal function. Our findings aligned with the current data which report the etiology of AKICS as multifactorial^[24-26]^, correlating with advanced age, preexisting kidney disease, and left ventricular impairment, which are some of the important predictors of AKI development^[20]^.

Decreased left ventricle EF and increased SCr levels are risk factors for major complications and high mortality after cardiac surgery; they also contribute to the development of postoperative AKI^[23]^. Indeed, as our findings demonstrate, severely decreased left ventricle EF is one of the strongest risk factors related to postoperative AKI.

Optimizing cardiac function could be a good strategy to minimize the risk of postoperative AKI, according to Leppikangas et al.^[27]^. They reported that the use of preoperative calcium sensitizer infusion could be a good strategy to improve left ventricular EF in patients with high-risk cardiac surgery^[27]^.

Coronary angiography with iodinated contrast media is usually performed before cardiac operations to define the extent and severity of CAD. The use of iodinated contrast media is associated with contrast-induced nephropathy, a common and important potential complication after coronary angiography^[28]^.

Another factor that influences the risk of AKI is the time elapsed between cardiac catheterization and CABG; in case of urgent surgeries, it is not possible to delay the surgery after the angiography. Recent reports focused on the time interval between coronary angiography and cardiac operation as the main determinant of postoperative renal failure^[29]^. The notion that a 'double hit' on renal function in close succession may increase the risk of AKI provides the basis for the recommendation to wait before exposing the kidneys to a second insult^[28]^. However, there is no consensus for a specific time delay between cardiac catheterization and CABG operation.

Mehta et al.^[30]^ recently reported that the risk of post-CABG AKI development was "inversely and modestly related to the time between cardiac catheterization and CABG", with the highest incidence occurring in patients operated on ≤1 day after cardiac catheterization. This contrasts with our findings; we found no statistical difference between the time interval from catheterization to CABG and the development of postoperative AKI^[30]^.

Identification and categorization of these high-risk patients allow for optimal decision-making for earlier interventions and better management strategies.

One of our main findings was that the use of CCB drugs has a possible nephroprotective effect in patients undergoing myocardial revascularization with extracorporeal circulation, similarly to the findings reported by Passaroni et al.^[31]^. They found that patients who did not receive CCB medications presented a higher percentage of AKI development after CABG with CPB. The nephroprotection effect is due to inhibition of glomerular vasoconstriction, producing vasodilatation of the arterioles and increasing the natriuretic effect^[31]^.

### Predictors

Preoperative, intraoperative, and postoperative periods are all important for the development of AKI, but the use of traditional markers such as SCr levels to diagnose AKI can limit and even delay its diagnosis^[32]^; there could already exist an acute renal lesion with a normal SCr value. This occurs in addition to the hemodilution in the first postoperative hours after cardiac surgery. Thus, there would not be an increase in SCr levels and the diagnosis of AKI could be delayed^[33]^.

To prevent CABG-related AKI, predictive risk scores based on preoperative variables have been developed, such as the Cleveland Clinic score, Mehta score, SRI score, AKICS score, and age, creatinine, EF (ACEF) score^[32]^. However, there is still no consensus to recommend the use of a specific score to predict AKI before CABG^[32]^.

The ACEF score was first described in 2009 in a publication for quick bedside evaluations^[34]^. According to Chen et al.^[23]^, "the ACEF score may be the best and easiest tool to guide preventive and early therapeutic strategies for AKI to improve patient clinical outcomes" with a ROC curve of 0.781±0.027 (95%CI, 0.729-0.834; *P*=0.001).

Moreover, we found that the combination of factors such as hypertension, EF <30, eGFR, EuroSCORE II, and lack of CCB intake could bring us a model with a better ROC curve (0.783±0.036; 95%CI, 0.713-0.854; *P*<0.001). This scoring system may be an applicable model to predict postoperative AKI development because it uses clinical data, which can be readily and rapidly obtained, making it very appropriate for elective or urgent surgery^[23]^. More effort is required to develop and validate different prediction models to identify postoperative AKI in an easy-to-use, accurate pattern.

## CONCLUSION

AKI following isolated CABG surgery occurs with some frequency and it is of great clinical importance, related with poor postoperative outcomes, prolonged ICU stay, and high mortality rates. Independent risk factors for developing postoperative AKI consist of age, hypertension, EF <30%, eGFR, EuroSCORE II, and no intake of CCB drugs. These factors provide an easy and accurate model to predict postoperative AKI in patients undergoing cardiac surgery.

**Table:** 

Authors' roles & responsibilities	
COL	Contribution to the design of the study; data collection; statistical analysis; discussion of results; manuscript writing; article review; final manuscript approval
MFM	Article review; final manuscript approval
LPD	Article review; final manuscript approval
PJM	Contribution to the design of the study; statistical analysis; article review; final manuscript approval
YC	Conception and design of the project; data collection; discussion of results; article review; final manuscript approval
